# Symptoms, side effects, quality of life and financial toxicity matter to patients with cancer, we need to do a better job of capturing and reporting these concepts in trials and clinical care

**DOI:** 10.1136/bmjonc-2025-000879

**Published:** 2025-06-17

**Authors:** Melanie Calvert, Roger Wilson, John Devin Peipert

**Affiliations:** 1 Centre for Patient Reported Outcomes Research, University of Birmingham, Birmingham, UK; 2 NIHR Birmingham Biomedical Research Centre, Birmingham, UK; 3 Birmingham Health Partners Centre for Regulatory Science and Innovation, University of Birmingham, Birmingham, UK; 4 NIHR Blood and Transplant Research Unit Precision Cellular Therapeutics, Birmingham, UK; 5 Patient Member Cancer Research Advocates Forum UK, London, UK

**Keywords:** Clinical Trial, Ethics

 For those experiencing a cancer diagnosis, survival and disease progression are at the forefront of minds. Alongside this, patients want to understand disease symptoms, potential side effects of therapies and impacts on their quality of life and ability to take part in social activities, education and work.

Patient-reported outcomes (PROs) are increasingly used in cancer trials to assess treatment efficacy and tolerability, and they can provide valuable insights to inform regulatory decision-making, reimbursement and clinical guidelines.^1 2^ Crucially, PROs can be used to provide information to patients and clinicians to make shared decisions about care. PROs are also increasingly used in routine clinical practice to monitor symptoms, function and quality of life over time, signalling when patients are in distress, optimising and prioritising visit timing, and identifying additional care needs.

Despite the oncology field being at the forefront of PROs research for many decades and recognition of the value of PROs being realised by regulators, health technology assessment (HTA) agencies, pharmaceutical companies, clinicians and patients, much still needs to be done to realise the promise of PROs to transform oncology care with meaningful impact for patients.^3^ Although PROs are collected in many thousands of patients with cancer each year, these data are often not reported or methodological issues such as missing data hamper interpretation.^4^ We need to get this right, it matters. In research, collecting PRO data and not reporting is unethical.^5^ In clinical care, collecting PROs and then not using this information in care is also potentially unethical and will jeopardise patient buy-in to completing PROs.^6^

It is therefore pleasing to see national momentum for change. In this issue, Puccini *et al* summarise expert consensus from the 21st Italian Association of Medical Oncology (AIOM) National Conference, emphasising the necessity of including PROs as endpoints in all phases of cancer care and clinical research.^7^ They identify electronic PROs and financial toxicity as emerging areas of importance, advocate for methodological standardisation and highlight practical and organisational challenges to implementation. They call for a more structured and widespread use of PROs in both research and practice and for healthcare systems to invest in digital infrastructure, provide clinician training in quality of life data interpretation and adopt policy frameworks that prioritise patient-centred metrics, including financial toxicity. If implemented well, such steps could drive a shift towards more responsive and equitable oncology care.^6 8^

Training is key, yet PROs are not widespread in the medical curricula, despite symptom and side effect management and improvement or maintenance of quality of life being a cornerstone of high-quality cancer care. Greater standardisation of PRO assessment and training of clinicians to use the data could radically shift care.^9^ Missing data and participant burden are often identified as major problems with PROs, as noted in the conference proceedings; however, these can be addressed.^6 10^ Crucial is to inform trial participants or patients in routine settings why their data is being collected, how it will be used and who will be accessing the results. We know that patients are more likely to respond to PROs if they know that the information gleaned from their reports will be used in care decisions.^6^ Selection of measures that address concepts that matter to patients with cancer is key. The US Food and Drug Administration core set provides a useful resource that can be used from early phase cancer trials through to later phase settings.^2^ ‘Burden’ is a frequent word associated with PROs, but in the context of other activities faced by patients with cancer in their treatment journey, one could argue a brief questionnaire is least burdensome of all. We should not be gatekeeping and overly paternalistic when these data can really inform care. More work needs to be undertaken to report PROs in a way that is accessible for patients and their loved ones to make informed decisions.

The Italian Association of Medical Oncology summary provides actions that can be implemented globally. Operationalising will be central to success.

## Data Availability

No data are available.
